# Next-generation antipsychotics- Trends and perspectives beyond dopaminergic and glutamatergic agents

**DOI:** 10.1192/j.eurpsy.2023.1177

**Published:** 2023-07-19

**Authors:** O. Vasiliu, A. G. Mangalagiu, B. M. Petrescu, C. A. Candea, C. Tudor, D. Ungureanu, M. Miclos, C. Florescu, A. I. Draghici, R. E. Bratu-Bizic, M. Dobre, A. F. Fainarea, M. C. Patrascu

**Affiliations:** Psychiatry Department, Dr. Carol Davila University Emergency Central Military Hospital, Bucharest, Romania

## Abstract

**Introduction:**

Three generations of antipsychotics, all of which are based on the dopaminergic hypothesis of schizophrenia, are available for clinical use. Still, more than 66% of the patients diagnosed with schizophrenia spectrum disorders (SSD) could not achieve remission. Also, the glutamatergic hypothesis of schizophrenia is supported by translational models of this disease, even if the antipsychotics derived from this conceptual framework are not yet available on the market. However, the need for new pathogenesis models for schizophrenia and new generations of antipsychotics is acute, therefore, an exploration of the antipsychotics in the pipeline could be helpful in understanding the current stage of research in schizophrenia.

**Objectives:**

To assess the evidence supporting the potential benefits of new antipsychotics in the pipeline.

**Methods:**

A literature review was performed through the main electronic databases PubMed, Cochrane, Clarivate/Web Of Science, and EMBASE) and clinical trials repositories (US National Library of Medicine and World Health Organization Clinical Trials Registry Platform) using the search paradigm “antipsychotics” AND “schizophrenia” AND “non-dopaminergic” AND “non-glutamatergic”. All papers published between January 2010 and September 2022 were included.

**Results:**

Xanomeline/trospium (xanomeline is a muscarinic M1/M4 receptor agonist at the central nervous system, while trospium limits its peripheral effects) was efficient for schizophrenia in one phase III clinical trial. Pimavanserin (a selective 5HT2A inverse agonist and antagonist) was efficient in improving negative symptoms of schizophrenia in a phase II trial. Roluperidone (a 5HT2A and σ2 receptor antagonist) has been associated with favorable results in phase III clinical trials, mainly on negative symptoms of schizophrenia. SEP-363856 is a TAAR-1 agonist and 5HT1A agonist, currently explored in phase III clinical trials for schizophrenia. MK-8189 is a phosphodiesterase 10A inhibitor, investigated in phase III clinical trials for schizophrenia.

**Conclusions:**

Based on the retrieved data in the literature, multiple mechanisms, other than glutamatergic and dopaminergic pathways, are currently being investigated, and many of the antipsychotics based on these mechanisms are in the advanced stage of research. This is important not only for the clinical need to find more efficient and tolerable drugs for patients with schizophrenia but also because they may shed new light on the pathogenesis of this disease.

**Disclosure of Interest:**

None Declared

